# Trends and disparities in aortic dissection mortality in the united states: a retrospective analysis

**DOI:** 10.1186/s12872-025-05446-5

**Published:** 2026-01-22

**Authors:** Mohamed Fawzi Hemida, Alyaa Ahmed Ibrahim, Nafila Zeeshan, Mohammad Rayyan Faisal, Krish Patel, Mirna Hussein, Arwa Khaled Dessouky, Maryam Saghir, Eshal Saghir, Muhammad Raza Sarfraz, Zahin Shahriar, Maha Al Haj Kadour, Abdullah Farahat Elbanna, Mohamed Ahmed Rahma Dawelbait, Muhammad Faizan Ali, Ahmad M. Abdelkhalek, Rana Sayed, Khaled Ali

**Affiliations:** 1 Faculty of Medicine, Alexandria University, Alexandria, Egypt; 2 Department of Medicine, Dow University of Health Sciences, Karachi, Pakistan; 3 C. U. Shah Medical College, Surendranagar, Gujarat India; 4 Department of Medicine, Jinnah Sindh Medical University, Karachi, Pakistan; 5 Allied Hospital, Faisalabad Medical University, Punjab, Pakistan; 6 Dhaka Medical College Hospital, Dhaka, Bangladesh; 7 Faculty of Medicine, Damascus University, Damascus, Syria; 8 Department of Medicine, Division of Rheumatology and Immunology, Vanderbilt University Medical Center, Nashville, TN USA; 9 Department of Internal Medicine, Faculty of Medicine, University of Khartoum, Khartoum, Sudan; 10 Jinnah Postgraduate Medical Center, Karachi, Pakistan; 11 Northwestern Medicine McHenry Hospital, Rosalind Franklin University, Chicago, USA; 12 Kasr Alainy Faculty of Medicine, Cairo University, Cairo, Egypt; 13 Internal Medicine Department, Rosalind Franklin University of Medicine and Science, North Chicago, Chicago, IL USA; 14 Faculty of Medicine, Alexandria University, Champollion street, Al Mesallah Sharq, Al Attarin, Alexandria Governorate, 5372066 Egypt

**Keywords:** Aortic dissection, Mortality, Trends, Cardiovascular

## Abstract

**Background:**

Aortic Dissection (AD) is a life-threatening condition and one of the major causes of death in the U.S. Despite its clinical significance, trends in AD related mortality remain understudied. We aim to analyze nationwide mortality trends in AD in the U.S.

**Methods:**

Data from CDC WONDER (1999–2024) identified U.S mortality rates in adults aged ≥ 25 years with AD (ICD-10: I71.0). Crude mortality rates (CMRs) and age-adjusted mortality rates (AAMRs) per 100,000 were calculated. Trends were analyzed using Joinpoint regression to estimate annual percent change (APC) and average annual percent change (AAPC).

**Results:**

From 1999 to 2024, a total of 115,449 deaths from AD were recorded. The AAMR increased from 2.1 in 1999 to 2.4 in 2024 (AAPC of 0.46; 95% CI: 0.31 to 0.61; *p* < 0.001). Men had higher mortality than women (Overall AAMR: 2.62; *p* < 0.001 vs. 1.45; *p* < 0.001) (AAPC: 0.22; 95% CI: 0.05 to 0.39 vs. 0.61; 95% CI: 0.42 to 0.83) respectively. Racially, non-Hispanic (NH) Blacks demonstrated the greatest overall AAMR of 3.11. Regionally, AAMRs were highest in the Midwest (2.17) followed by the West (2.14). The urban areas had higher overall AAMRs than the rural areas (1.95, *p* = 0.27 vs. 1.90, *p* = 0.074). The majority of deaths occurred in medical facilities (88,976 deaths, 77.06%) and in older adults with CMR (5.8).

**Conclusion:**

Mortality trends in AD increased from 1999 to 2024. Higher trends occurred in men, urban areas, Midwest region, NH Black population and medical facilities.

**Graphical Abstract:**

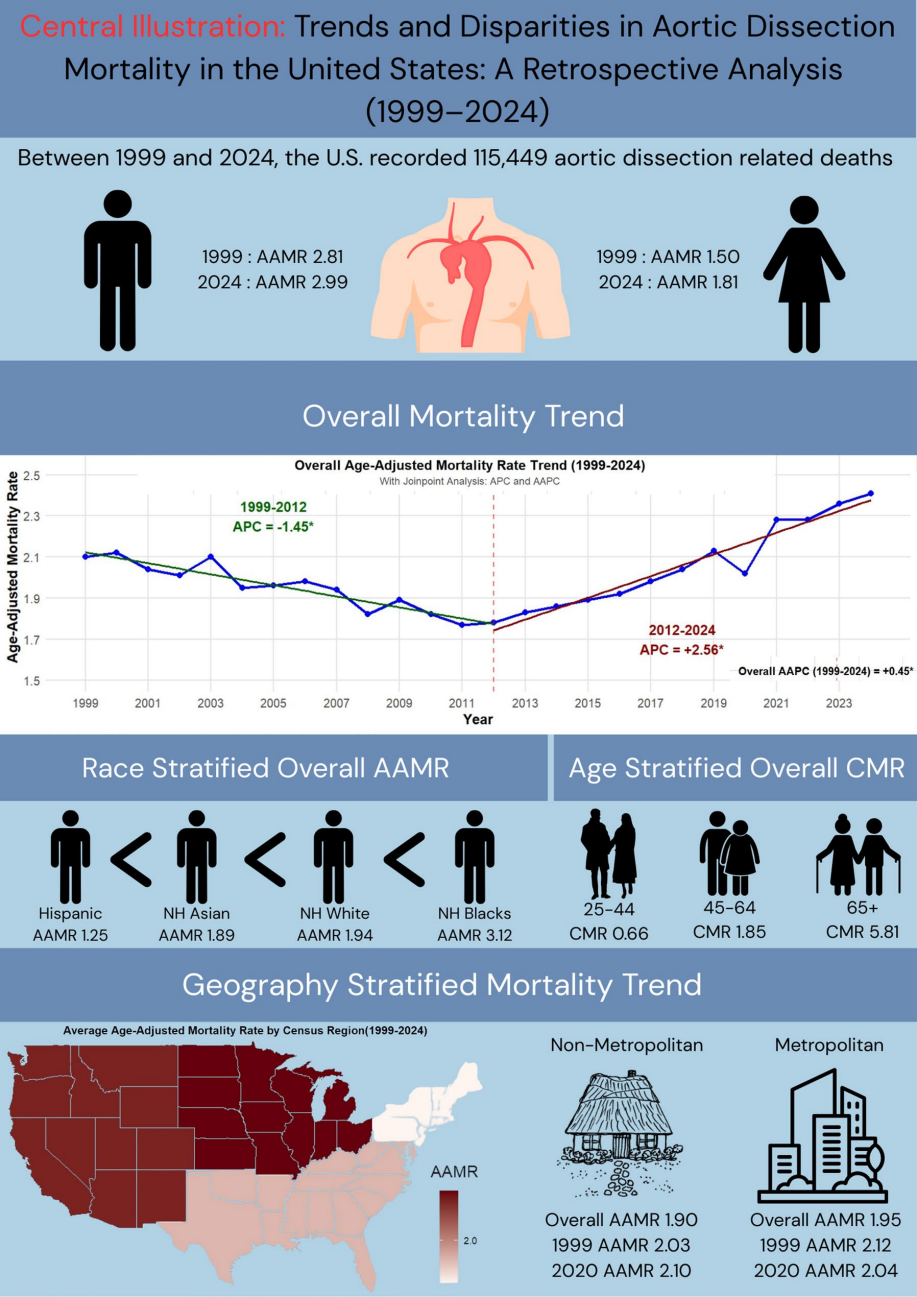

**Supplementary Information:**

The online version contains supplementary material available at 10.1186/s12872-025-05446-5.

## Introduction

Aortic dissection (AD) is the most common acute aortic disease with high morbidity and mortality requiring emergent diagnosis and surgical treatment if indicated. Surgical outcomes have improved, with contemporary survival to discharge at Centers of Excellence of 85% to 90%. [1] Therefore, AD should be considered in the differential diagnosis of any patient with chest, back, or abdominal pain. [2] About 67% of patients presented with type A AD, with the remaining 33% type B. Two-thirds of the included patients were men, with a mean age of 63 years. [3] Some contributing risk factors are modifiable, such as hypertension, smoking, and dyslipidemia, requiring prompt diagnosis and treatment to reduce the incidence and progression of AD. [4]

The most accurate estimate of incidence comes from population-based studies. Between 1980 and 2015, population-based studies across Europe and North America reported an annual incidence ranging from 2.5 to 15 per 100,000 people. [5] Overall, patients with AD are at high risk for an adverse outcome and need to be managed aggressively in the hospital and long term with frequent follow-up. [6, 7] Untreated AD is a fatal condition, with an estimated mortality rate of 40% on initial presentation; this rate, however, increases by 1% every hour, and can reach an annual mortality rate of up to 90%. [8] Survival is related to prompt treatment, preexisting medical comorbidities, presence or absence of end-organ malperfusion, extent of aortic repair required, and the development of postoperative complications. [9]

Furthermore, Acute AD is a life-threatening emergency condition associated with high morbidity and mortality rates. [10] The mortality rate is high because the condition is so frequently misdiagnosed. [11, 12] Prior studies have focused on the pathophysiology and treatment of AD, leaving a knowledge gap regarding the nationwide epidemiological trends in mortality. In this study, we aim to analyze U.S. mortality trends related to AD from 1999 to 2024 using data from the Centers for Disease Control and Prevention Wide-ranging Online Data for Epidemiologic Research (CDC WONDER) database. We specifically examine differences by age, sex, census region, and urbanization status to identify disparities and evolving trends over time.

## Methods

### Study setting and population

This retrospective analysis uses death certificate data retrieved from the CDC WONDER database and analyzes data for adults aged 25 and older to assess AD-related mortality from 1999 to 2024. We chose ≥ 25 years to focus on mature adult mortality patterns and excluded individuals younger than 25 years and documents with missing geographic and demographic data as AD risks differ substantially in younger populations. Diagnostic coding was employed using the International Statistical Classification of Diseases and Related Health Problems-10th Revision (ICD-10) with code: I71.0 for AD. Adults were defined as those who were ≥ 25 years at the time of death. Consequently, this age cutoff has been used by previous studies to define adults. [13] The primary outcome of this study was AD-related mortality. This was identified using publicly available Multiple Cause-of-Death mortality data from the CDC WONDER database. Deaths were included if AD (ICD-10 code I71.0) was listed anywhere on the death certificate, either as the underlying cause of death or as one of the contributing causes of death. This comprehensive approach ensures the capture of all deaths where AD played a documented role, regardless of its position on the death certificate. Institutional review board approval was not required for this study as it used de-identified public use data provided by the government and adhered to the strengthening the reporting of observational studies in epidemiology (STROBE) guidelines for reporting. [14]

### Data abstraction

Data for population size, year and demographic variables (sex, age, race, region and states) were extracted. Place of Death was categorized as medical facility, hospice, home, or nursing home/long-term care facility. Race and ethnicity were classified as Non-Hispanic (NH) White, NH Black or African American, Hispanic or Latino, NH American Indian or Alaskan Native, and NH Asian or Pacific Islander. The National Center for Health Statistics Urban-Rural Classification Scheme was used to assess the population by urban (metropolitan areas) or rural (non-metropolitan areas), based on the 2013 U.S. Census classification. [15, 16] It is important to note that urban-rural data were consistently available and analyzed only for the period 1999–2020 due to historical limitations in WONDER stratifications and a prior published cardiovascular paper has referred to this issue. [17] Regions were stratified as Northeast, Midwest, South, and West according to the U.S. Census Bureau definitions. Age groups were divided into 3 groups (25–44, 45–64, and ≥ 65).

### Statistical analysis

Crude mortality rates (CMRs) and age adjusted mortality rates (AAMRs) per 100,000 population from 1999 to 2024 by year, sex, race/ethnicity, state, and urban-rural status, for the years 1999 to 2020 only, with 95% CIs were calculated, using the 2000 U.S. population as the standard. [18] CMR was determined by dividing the number of AD patients by the corresponding U.S. population of that year. It’s worth mentioning that we used CMRs exclusively for age-specific analyses, as age adjustment would mask true differences between age groups. For all other variables, AAMRs were calculated to ensure comparability across populations with differing age structures. The Joinpoint Regression Program (Joinpoint V 5.4.0.0, National Cancer Institute) was used to determine the average annual percent change (AAPC) and the annual percent change (APC) with 95% CI in AAMR to quantify national annual trends in AD related mortality. Joinpoint regression, a segmented regression technique, was used to identify points of trend change (join points) by fitting log-linear models and it used permutation tests to select the optimal number of join points (with maximum 3 joinpoints allowed), ensuring model fit. [19] Using this regression analysis, we identified the joinpoints, dividing the study period into segments based on observed inflection points. This method allows identification of significant changes in AAMR over time by fitting log-linear regression models where temporal variation occurred. APCs were considered increasing or decreasing if the slope describing the change in mortality was significantly different from zero using two tailed t testing. A p-value < 0.05 was considered statistically significant.

## Results

### Overall mortality trends

From 1999 to 2024, a total of 115,449 deaths from AD were recorded. Majority of deaths occurred in medical facilities (77.06%), followed by the decedent’s residence (14.59%), nursing homes/long-term care facilities (2.59%), and hospice settings (1.90%). A small proportion of deaths occurred in other locations (3.60%), while the remainder of deaths (0.24%) had an unknown place of death. (Supplemental Tables 1, 2).

The AAMR observed an overall significant rise during the study period, from 2.10 in 1999 to 2.41 in 2024, with an AAPC of 0.46 (95% CI: 0.31 to 0.61; *p* < 0.001).

**Fig. 1:**
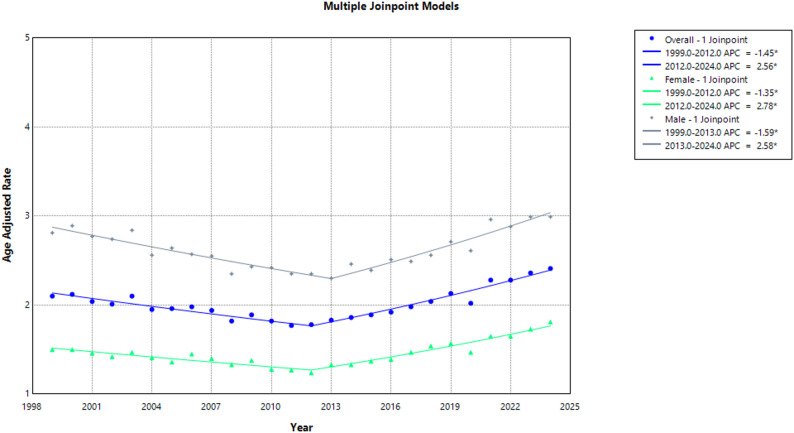
Overall and Sex-Stratified Aortic Dissection-Related AAMRs per 100,000 in Adults in the United States, 1999–2024

Time specific analysis showed a decline from 1999 to 2012, AAMR exhibited a significant decrease, from 2.10 to 1.78 (APC: −1.45; 95% CI: −1.90 to −1.07; *p* < 0.001), followed by a significant rise to 2.41 in 2024 (APC: 2.56; 95% CI: 2.16 to 3.08; *p* < 0.001). (Supplemental Tables 3, 4) (Fig. 1**)**.

### Mortality trends by gender

Men exhibited higher mortality than women (67,693 vs. 47,756 deaths). Correspondingly, the AAMR remained consistently higher in men, with overall AAMRs of 2.62 and 1.45 for men and women, respectively. In 1999, the AAMR was 2.81 among men and 1.50 among women. These values surged to 2.99 and 1.81, respectively, by 2024. However, women exhibited a greater AAPC of 0.61 (95% CI: 0.42 to 0.83; *p* < 0.001), compared to men with an AAPC of 0.22 (95% CI: 0.05 to 0.39; *p* = 0.011).

Time specific temporal trends showed a significant decrease in AAMR among women, from 1.50 in 1999 to 1.24 in 2012 (APC: −1.35; 95% CI: −1.92 to −0.83; *p* < 0.001), followed by a marked increase to 1.81 in 2024 (APC: 2.78; 95% CI: 2.30 to 3.40; *p* < 0.001).

AAMR among men showed a significant decrease from 2.81 in 1999 to 2.30 in 2013 (APC: −1.59; 95% CI: −2.01 to −1.22; *p* < 0.001), followed by a significant increase to 2.99 in 2024 (APC: 2.58; 95% CI: 2.10 to 3.16; *p* < 0.001). (Supplemental Tables 1, 3, 4) **(**Fig. 1**)**.

### Trends in mortality by Race/Ethnicity

When stratified by race, NH White individuals accounted for the highest number of deaths (84,746), followed by NH Black/African American individuals (18,705), Hispanic/Latino individuals (6,914), and NH Asian/Pacific Islander (4,570). However, the overall AAMR was highest among NH Blacks (3.12), followed by NH Whites (1.94), NH Asians (1.89), and Hispanic/Latino individuals (1.25).

All races observed an increase in AAMR between 1999 and 2024; 2.05 to 2.28 (AAPC: 0.38; 95% CI: 0.21 to 0.56; *p* = 0.004) among NH White individuals, 2.89 to 4.16 (AAPC: 1.57; 95% CI: 1.08 to 2.20; *p* < 0.001) among NH Blacks, 1.34 to 1.56 (AAPC: 0.19; 95% CI: −0.32 to 0.83; *p* = 0.361) among Hispanic/Latino individuals, and 1.84 to 1.85 (AAPC: −0.64; 95% CI: −1.19 to 0.04; *p* = 0.064) among NH Asians.

**Fig. 2:**
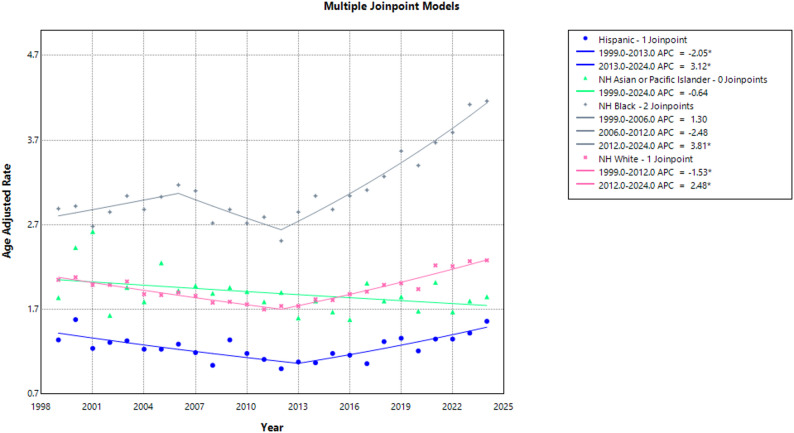
Aortic Dissection-Related AAMRs per 100,000 Stratified by Race in Adults in the United States, 1999–2024

Time specific trends showed that AAMR among Hispanics decreased significantly from 1.34 in 1999 to 1.08 in 2013 (APC: −2.05; 95% CI: −4.09 to −0.72; *p* = 0.003), and followed by a significant increase, reaching 1.56 in 2024 (APC: 3.12; 95% CI: 1.83 to 5.81; *p* < 0.001). No joinpoints were detected for NH Asians. NH Black individuals demonstrated an initial non-significant increase in AAMR from 2.89 in 1999 to 3.17 in 2006 (APC: 1.30; 95% CI: −0.63 to 7.52; *p* = 0.21), followed by a non-significant decrease to 2.51 in 2012 (APC: −2.48; 95% CI: −7.84 to 7.65; *p* = 0.21), and a subsequent significant increase to 4.16 in 2024 (APC: 3.81; 95% CI: 1.28 to 5.94; *p* = 0.004). NH White individuals experienced a significant decline in AAMR from 2.05 in 1999 to 1.74 in 2012 (APC: −1.53; 95% CI: −2.00 to −1.10; *p* < 0.001), followed by a significant upward trend, reaching 2.28 in 2024 (APC: 2.48; 95% CI: 2.02 to 3.03; *p* < 0.001). (Supplemental Tables 1, 3, 5)** (**Fig. 2**)**.

### Age-Specific mortality trends

From 1999 to 2024, highest mortality was achieved in older adults (66,915 deaths), compared to middle-aged adults (37,605 deaths), and young adults (10,929 deaths). Consequently, older adults achieved the greatest overall CMR (5.81), followed by middle-aged adults (1.85), and young adults (0.50). Among young adults, CMR showed an increase between 1999 and 2024; 0.42 to 0.65 (AAPC: 1.85; 95% CI: 1.38 to 2.32; *p* < 0.001). Similarly, CMR among middle-aged adults increased from 1.77 to 2.31 (AAPC: 1.23 95% CI: 0.98 to 1.53; *p* < 0.001) throughout the same period. However, older adults showed a decrease in CMR from 6.63 in 1999 to 6.40 in 2024 (AAPC: −0.36; 95% CI: −0.56 to −0.14; *p* = 0.003).

**Fig. 3:**
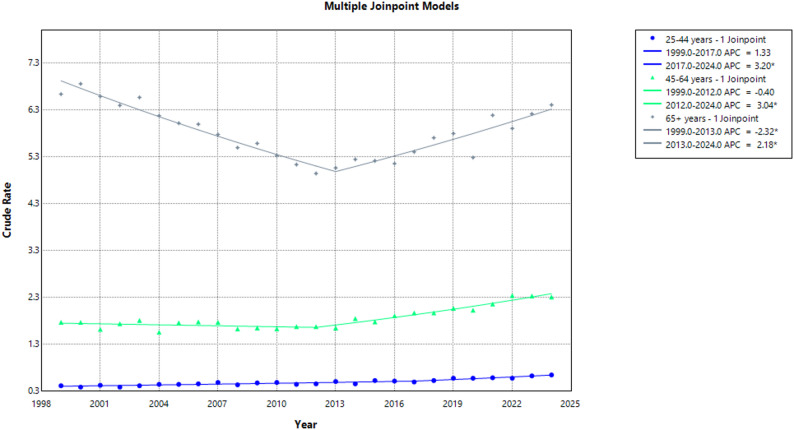
Aortic Dissection-Related CMRs per 100,000 Stratified by Age in Adults in the United States, 1999–2024

Time specific temporal trends showed a non-significant increase in CMR among young adults, from 0.42 in 1999 to 0.50 in 2017 (APC: 1.33; 95% CI: −1.24 to 3.15; *p* = 0.1), followed by a significant increase to 0.65 in 2024 (APC: 3.20; 95% CI: 1.57 to 8.88; *p* = 0.023). Middle-aged adults saw an initial non-significant decrease in CMR from 1.77 in 1999 to 1.68 in 2012 (APC: −0.40; 95% CI: −1.42 to 0.26; *p* = 0.23). This was followed by a significant increase to 2.31 in 2024 (APC: 3.04; 95% CI: 2.40 to 4.08; *p* < 0.001). Older adults showed variation in CMR, initially decreasing significantly from 6.63 in 1999 to 5.06 2013 (APC: −2.32; 95% CI: −2.90 to −1.82; *p* < 0.001), and then rising significantly, reaching 6.40 in 2024 (APC: 2.18; 95% CI: 1.57 to 2.99; *p* < 0.001). (Supplemental Tables 3, 6)** (**Fig. 3**)**.

### Regional trends

Between 1999 and 2024, the Southern region reported the highest number of deaths (40,616), followed by the Midwest (27,456), the West (27,194), and the Northeast (20,183). Overall, AAMRs were highest in Midwest (2.17), West (2.15), the South (1.93), and the Northeast (1.83). All regions demonstrated an overall increase in AAMR throughout the study period. In the Northeast, AAMR increased from 2.00 to 2.12 (AAPC: 0.29; 95% CI: −0.05 to 0.65; *p* = 0.09), while the Midwest saw an increase from 2.40 to 2.59 (AAPC: 0.57; 95% CI: 0.34 to 0.82; *p* < 0.001). The Southern region observed an increase from 1.92 to 2.43 (AAPC: 0.86; 95% CI: 0.47 to 1.34; *p* = 0.004), while the West observed an increase from 2.22 to 2.42 (AAPC: 0.30; 95% CI: −0.09 to 0.72; *p* = 0.12) over the same period.

Time specific trends showed that the AAMR in the Northeast saw an initial significant decline from 2.00 in 1999 to 1.55 in 2013 (APC: −1.44; 95% CI: −2.57 to −0.70; *p* < 0.001), followed by a significant increase to 2.12 in 2024 (APC: 2.55; 95% CI: 1.56 to 4.46; *p* < 0.001). In the Midwest, AAMR dropped significantly from 2.40 in 1999 to 1.88 in 2011 (APC: −1.45; 95% CI: −2.32 to −0.80; *p* < 0.001), subsequently increasing significantly reaching 2.59 in 2024 (APC: 2.46; 95% CI: 1.92 to 3.23; *p* < 0.001).

**Fig. 4:**
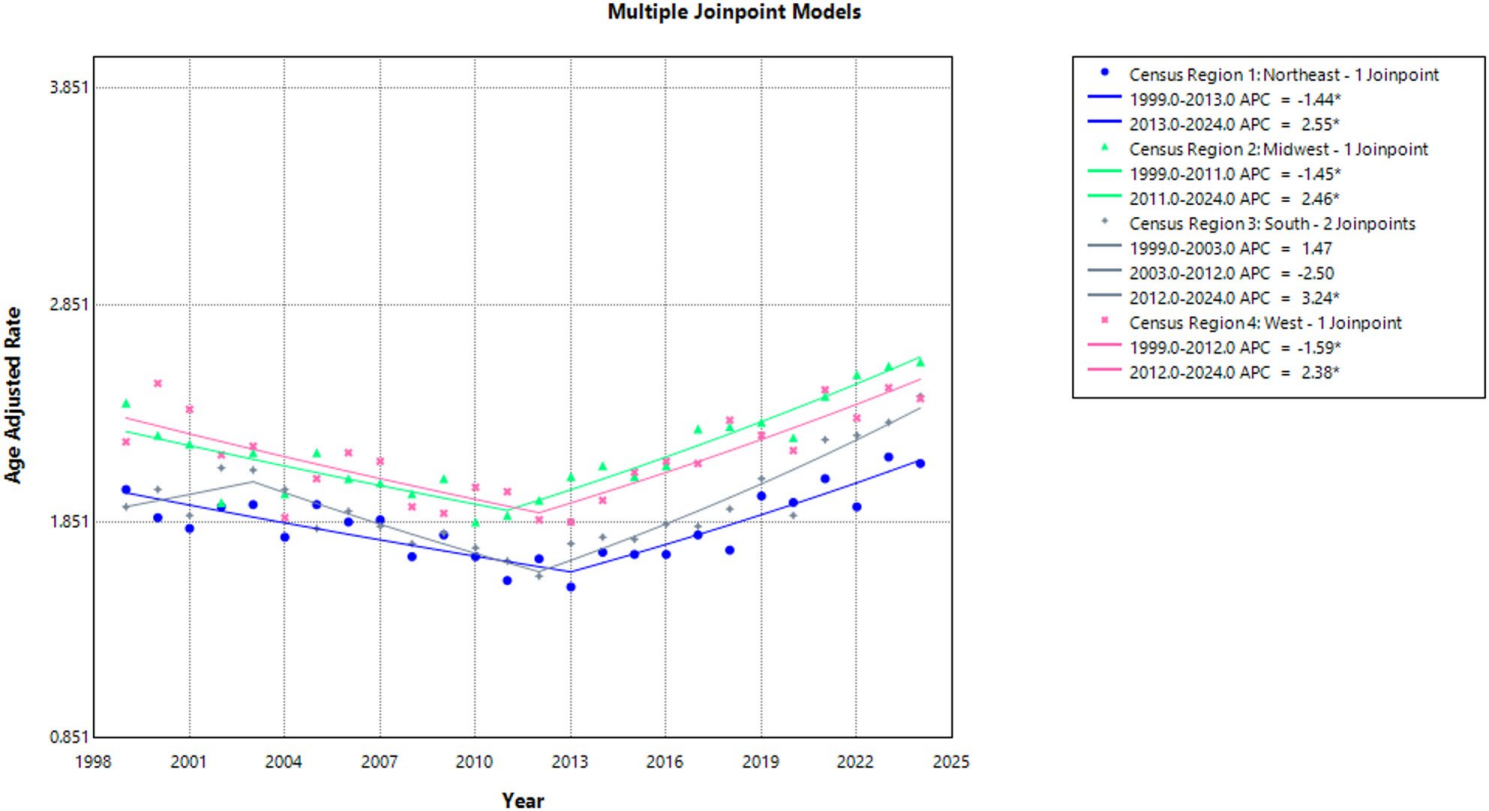
Aortic Dissection-Related AAMRs per 100,000 Stratified by Census Region in Adults in the United States, 1999–2024

In the Southern region, AAMR rose non-significantly, from 1.92 in 1999 to 2.09 in 2003 (APC: 1.47; 95% CI: −1.64 to 8.75; *p* = 0.39), followed by a non-significant decrease to 1.60 in 2012 (APC: −2.50; 95% CI: −6.39 to 4.92; *p* = 0.15), and a steady yet significant rise, reaching 2.43 in 2024 (APC: 3.24; 95% CI: 2.17 to 4.45; *p* = 0.003). The Western region experienced a marked decrease in AAMR from 2.22 in 1999 to 1.86 in 2012 (APC: −1.59; 95% CI: −3.26 to −0.65; *p* = 0.002), followed by a significant rise, reaching 2.42 in 2024 (APC: 2.38; 95% CI: 1.40 to 3.95; *p* < 0.001). (Supplemental Tables 3, 7)** (**Fig. 4**)**.

### Urbanization disparities

Between 1999 and 2020, metropolitan areas accounted for a higher mortality (76,787 deaths) compared to non-metropolitan areas (14,922 deaths). The overall AAMR remained higher in metropolitan areas (1.95) relative to non-metropolitan areas (1.90).

From 1999 to 2020, metropolitan areas demonstrated a decrease in AAMR, from 2.12 to 2.04 (AAPC: −0.15; 95% CI: −0.39 to 0.13; *p* = 0.274), compared to non-metropolitan areas, where AAMR rose from 2.03 to 2.10 (AAPC: 0.34; 95% CI: −0.05 to 0.74; *p* = 0.074) over the same period.

**Fig. 5:**
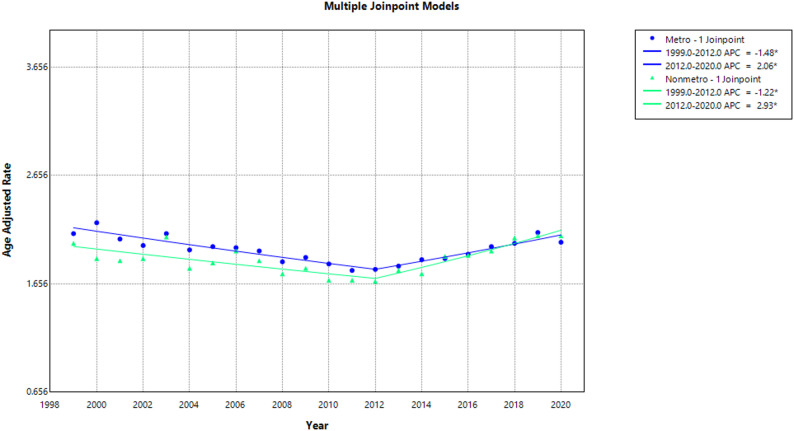
Aortic Dissection-Related AAMRs per 100,000 Stratified by Urban-Rural status in Adults in the United States, 1999–2020

Segmented trend analysis (1999–2020) showed that metropolitan regions experienced a significant decline in AAMR from 2.12 in 1999 to 1.79 in 2012 (APC: −1.48; 95% CI: −2.14 to −0.99; *p* < 0.001), followed by a significant increase to 2.04 in 2020 (APC: 2.06; 95% CI: 1.20 to 3.61; *p* < 0.001). In non-metropolitan areas, AAMR decreased significantly from 2.03 in 1999 to 1.68 in 2012 (APC: −1.22; 95% CI: −2.27 to −0.57; *p* < 0.001), followed by a significant upward trend, reaching 2.10 in 2020 (APC: 2.93; 95% CI: 1.64 to 5.51; *p* < 0.001). (Supplemental Tables 3, 8) (Fig. 5**)**.

### States mortality trends

States that classified in the top 90th percentile, namely Colorado, Delaware, District of Columbia, Hawaii, and Washington, had nearly twice the AAMRs as compared to states in the lower 10th percentile i.e. Connecticut, Kentucky. Maine, Massachusetts, and New Jersey. (Supplemental Table 9).

## Discussion

We analyzed mortality trends of AD across demographics in the U.S over 25 years. We have several important findings to note. First, mortality has overall increased, with a substantial increase from 2012 to 2024. Second, men experienced higher mortality rates than women; however, women had a sharper rise in mortality post-2012. Third, NH black or African American individuals had the highest AAMR. Moreover, ≥ 65 older adults had the highest CMRs, reflecting the heavy mortality burden. Lastly, the rural Midwest was most disproportionately affected. AD is a life-threatening situation that can compromise vessels, leading to ischemia, aortic regurgitation, pericardial tamponade, and malperfusion of visceral, spinal, or renal arteries. Chronic hypertension is present in 70–75% of cases, making it the single most common risk factor. [20] AD accounts for 3 out of every 1000 emergency department presentations. [21] Given its catastrophic complications and persistently high fatality, the burden of AD remains alarming despite medical advancements.

Our analysis showed that AD mortality decreased from 1999 to 2012, after which it reversed and began rising steadily. Our trends align with recent U.S. population studies. Nazir et al. (1999–2019) documented a decline in AAMR through 2012, followed by a rise into 2019. [22] Our analysis (1999–2024) confirms and extends this resurgence, with detailed stratification across age, sex, race, region, urbanization, place of death, and metropolitan status. Compared with Nazir et al., we extend surveillance through 2024, demonstrating that the post-2012 rise persisted beyond 2019.

The initial decline is supported by several studies present in the current literature. For instance, Mody et al. reported a decline in 30-day mortality after acute AD among Medicare beneficiaries from 30.7% in 2000 to 21.4% in 2011 for Type A surgical repair and 24.9% to 21.0% for Type B. [23] Furthermore, Zimmerman et al. also noted an improvement in in-hospital mortality through 2012. However, contemporary hospitalization-based evaluations beyond 2012 are very limited. Consequently, the marked post-2012 mortality increase we observed has not been fully explored in studies of admissions or treatment patterns, which may have contributed to shifts in case detection, reporting, or outcomes. [24]

These observed initial, declining trends have several plausible explanations. First, advances in diagnosis and treatment, such as the adoption of safer open surgical strategies like antegrade cerebral perfusion, root- and valve-sparing procedures, and expanded arch replacement, may have played a significant role in reducing postoperative mortality. At the same time, rapid uptake of Thoracic Endovascular Aortic Repair (TEVAR) for complicated Type B dissections, and CT angiography becoming widely available, improved early detection and treatment. [3, 25, 26] Furthermore, better education on blood pressure control, improved lipid management, and the introduction of antihypertensive medications and statins have all contributed to the overall decline. [27, 28] Lastly, insurance expansion and increased healthcare access allowed patients to get screened and monitored for any early signs to be treated promptly. Collectively, all these factors may have contributed significantly to the overall decline; however, due to the retrospective nature of the CDC database, we can’t infer cause and cause-and-effect relationship.

Conversely, our analysis also observed a pronounced increase post-2012. Muntner et al. (2020) showed that U.S. hypertension control plateaued and then worsened from 2014 to 2017. [29] With hypertension being a known risk factor, the decline in blood pressure control might have contributed to the rising dissection deaths. Moreover, population-level increase in obesity, hyperlipidemia, diabetes, and stimulant use, well documented in U.S. epidemiological studies, may have further amplified the underlying risk for AD. [30–32] The recent increase may reflect multifactorial effects, including reduced healthcare access and worsened hypertension control. For instance, U.S. emergency department visits declined by 42%, which may have led to increased pre-hospital mortality due to delayed diagnosis and treatment. [33] Moreover, national cardiac surgery data also showed 30% fewer Acute Type A Aortic Dissection (ATAAD) repairs during the first surge, which highlights delayed treatment that has led to increased mortality. [34] Concurrently, multiple U.S. cohorts reported challenges in blood pressure control and disruptions to routine care, making people more vulnerable to AD. [35] Emerging data also indicate increased use of stimulants such as cocaine and methamphetamine. These agents are well-documented precipitants of acute AD due to their ability to induce abrupt surges in blood pressure, sympathetic activation, and severe vasospasm. [36] These contextual factors may have contributed to increased mortality, but this interpretation should be viewed as hypothesis-generating rather than causal due to the retrospective nature of the CDC WONDER dataset.

Our analysis reported males having higher AAMRs than females. Men are at higher risk of developing AD than women; however, women present later in the disease with worse outcomes. [20, 37] These differences may be explained by risk profiles, as men are more likely to have hypertension, heritable aortic disease, and atherosclerosis, and engage in high-risk behaviors such as smoking. [38] Women, in contrast, tend to be older at presentation and may experience worse outcomes due to delayed diagnosis, greater comorbidity, age-related stiffening of vessels, and loss of estrogen’s protective effect. [39] Furthermore, there are also discrepancies between men and women in society guidelines for abdominal aortic aneurysm (AAA) screening, which contributes to the late presentation in women. [40] In addition to that, pregnancy is also a recognized risk factor, occurring in about 0.0004% of pregnancies, due to hemodynamic stress, hormonal weakening of connective tissue, and peripartum vascular strain. [41] While these mechanisms are plausible, our analysis cannot confirm them due to a lack of clinical detail.

Our analysis demonstrated that NH Black individuals had a persistently higher rate of AD mortality throughout the study period. Several plausible reasons can explain the vast growing racial disparity. African Americans are disproportionately affected by hypertension, the single strongest risk factor for dissection, as well as higher rates of atherosclerosis, diabetes, smoking, and kidney disease. [42] Moreover, vascular differences such as stiffer central arteries and a smaller aortic root diameter have also been associated with African ancestry. [43, 44] In addition to that, African American patients present with type A AD at a younger median age (54–55 years) compared to Whites (64–65 years), reflecting cumulative early risk exposure. Socioeconomic disadvantage (SED) compounds this risk; one study noted that 54% of African Americans admitted for ATAAD were from the lowest household income quartile. [45] Furthermore, African Americans are less likely to receive transcatheter valve replacement or referral to cardiac surgery than Whites. [46] Collectively, systemic inequities and biological risk both contribute to the growing burden among African Americans.

Our age-stratified analysis found adults aged ≥ 65 years to have the highest average CMR when compared to middle and younger adults. However, younger adults saw a pronounced increase in overall AAPC compared to middle-aged and older adults, who experienced an overall decline. These findings are consistent with the literature showing that 75% of ADs occur in adults aged ≥ 65. [20] This can be explained by the physiological weakening of the vascular wall. Aging also stiffens the aorta, raising pulse pressure and accelerating medial degeneration. [47] Comorbidities further increase mortality risk in surgical candidates. The sex gap narrows with age as women lose estrogen’s vascular protective effects. [48] With the aging population in the U.S., this represents an important concern for prevention and monitoring. Interestingly, we also found an overall rise in younger adults compared to the initial declines in middle-aged and older adults. This may be related to an increase in stimulant use and high-risk behaviors such as smoking and alcohol consumption in the younger population. [49] A case report of six younger male patients with a history of methamphetamine use has concluded that methamphetamine is associated with high rates of AD. [50] Furthermore, rising obesity and metabolic syndrome make younger individuals more susceptible to hypertension, which in turn raises the risk of AD. [51] Finally, genetic predisposition to diseases such as Marfan syndrome, Ehlers-Danlos syndrome, and bicuspid aortic valve also accounts for a significant proportion of AD in younger adults. [52] The alarming increase in mortality in the younger demographic raises concerns and calls for public health initiatives to integrate screening, prevention, and education to prevent any avoidable dissection.

Ultimately, our analysis revealed significant geographic disparities. The South accounted for the highest mortality burden overall, but the Midwest had the highest average AAMRs, with states such as Colorado, Delaware, the District of Columbia, and Hawaii disproportionately affected. Higher mortality in Western and Midwestern states may be linked to higher rates of hypertension and methamphetamine use, which have been strongly associated with AD and worse in-hospital outcomes even after controlling comorbidities. [53, 54]

In terms of urbanization, metropolitan areas had higher mortality than non-metropolitan areas; however increase after 2012 was more pronounced in non-metropolitan areas, highlighting widening urban-rural disparities. Tabassum et al. also reported higher AD-related mortality among hypertensive patients. The higher AAMR observed might be due to better diagnosis/reporting in urban centres, referral of complex cases, and concentration of high-risk lifestyle factors. [13] The post-2012 rising AD mortality in rural areas is concerning, likely reflecting limited healthcare access, delayed diagnosis, lack of specialized centres, and longer travel times to medical facilities. [55, 56] Moreover, people from lower socioeconomic status (SES) experience reduced short-term and long-term survival after AD. That’s because people living in (SED) being associated with increased comorbidities, such as hypertension and tobacco use, which again increases the risk of AD. [57]

### Limitations

This study has several limitations. First, reliance on ICD-10 death certificate coding may introduce misclassification, particularly given the inability of the CDC WONDER database to distinguish between AD type A and type B subtypes, as exclusively the specific ICD-10 code available (I71.0) aggregates all acute AD into a single category, or to capture sudden out-of-hospital deaths, potentially leading to underestimation of true mortality.

Second, the database lacks information on diagnostic transitions (e.g., expanded use of CT angiography), coding practice changes, or the timing of guideline updates; these unmeasured factors may have contributed to reporting variation, especially in earlier years, and could partly explain the recent rise through improved case recognition. Third, the absence of granular clinical variables, including blood pressure control, aortic dimensions, connective tissue disorders, bicuspid aortic valve, stimulant use, smoking, obesity, and other risk factors, limits our ability to explore mechanisms behind subgroup differences, such as higher mortality among African Americans, recently younger adults, and rural populations. Fourth, socioeconomic factors, insurance status, access to high-volume centers, and treatment-related variables (e.g., transfer delays, operative timing, or perioperative management) are not captured, precluding adjustment for key confounders and preventing assessment of care pathways. Moreover, mortality rates for certain racial groups were suppressed in CDC WONDER because of small cell counts, preventing meaningful analysis of these populations. Additionally, because of the suppressed data, we were not able to determine the place of death of one patient. That’s why our place of death data was available for only 115,448 deaths while the total number of deaths was 115,449.

Lastly, as an ecological, population-level analysis, the study cannot establish causality or evaluate individual-level associations, underscoring the need for future work integrating clinical and mortality datasets to enhance surveillance accuracy.

Future research should prioritize adherence to the 2022 ACC/AHA guidelines may contribute to continued reductions in AD incidence, the development of endografts that preserve collateral flow, the identification of biomarkers such as D-dimer or microRNAs for rapid diagnosis, and genetic studies to uncover additional risk factors. [58, 59].

## Conclusion

AD remains a life-threatening vascular emergency with a disproportionate burden among elderly men, African Americans, and individuals living in Western states. While overall mortality has modestly increased in the U.S. since 1999 in older adults and urban residents, recent years have shown an upward trend, particularly concerning, African American men, younger adults and rural residents. Addressing these disparities requires targeted public health strategies, including community-based blood pressure management programs, screening of high-risk populations with hypertension or heritable syndromes, and improved access to surgical and endovascular care.

## Supplementary Information

Supplementary Material 1.

## Data Availability

The data that support the findings of this study are openly available in CDC-WONDER at [https://wonder.cdc.gov/](https:/wonder.cdc.gov). The data supporting the findings of this study were obtained from the CDC WONDER online database (Centers for Disease Control and Prevention Wide-ranging Online Data for Epidemiologic Research). Further inquiries can be directed to the corresponding author.

## Abbreviations

AD: Aortic Dissection
CDC: Centers for Disease Control and Prevention
WONDER: Wide-Ranging Online Data for Epidemiologic Research
ICD-10: International Statistical Classification of Diseases and Related Health Problems, 10th Revision
STROBE: Strengthening the Reporting of Observational Studies in Epidemiology
NH: Non-Hispanic
U.S.: United States
CMR: Crude Mortality Rate
AAMR: Age-Adjusted Mortality Rate
CI: Confidence Interval
AAPC: Average Annual Percent Change
APC: Annual Percent Change
NCHS: National Center for Health Statistics
ATAAD: Acute Type A Aortic Dissection
AAA: Abdominal Aortic Aneurysm
SES: Socioeconomic Status
SED: Socioeconomic Disadvantage
ACC: American College of Cardiology
AHA: American Heart Association
