# Supplementary material for: Trends and disparities in aortic dissection mortality in the united states: a retrospective analysis
Source: BMC Cardiovasc Disord. 2026 Jan 22;26:66. doi: 10.1186/s12872-025-05446-5 (PMC12825201; doi:10.1186/s12872-025-05446-5)
Supplement: Supplementary file 1 — Supplementary Material 1. [file 12872_2025_5446_MOESM1_ESM.docx]

**Supplemental Table 1** Aortic Dissection–related Deaths, Stratified by Sex and Race, in Adults in the United States, 1999 to 2024.

| Year | Overall | Female | Male | NH Asian or Pacific Islander | NH Black or African American | NH White | Hispanic or Latino |
| --- | --- | --- | --- | --- | --- | --- | --- |
| 1999 | 3,736 | 1,548 | 2,188 | 85 | 487 | 3,014 | 143 |
| 2000 | 3,827 | 1,581 | 2,246 | 115 | 495 | 3,035 | 171 |
| 2001 | 3,732 | 1,542 | 2,190 | 131 | 476 | 2,960 | 150 |
| 2002 | 3,757 | 1,525 | 2,232 | 89 | 521 | 2,970 | 164 |
| 2003 | 3,952 | 1,582 | 2,370 | 114 | 567 | 3,085 | 175 |
| 2004 | 3,715 | 1,554 | 2,161 | 113 | 547 | 2,864 | 182 |
| 2005 | 3,858 | 1,550 | 2,308 | 143 | 593 | 2,927 | 184 |
| 2006 | 3,945 | 1,628 | 2,317 | 129 | 633 | 2,963 | 211 |
| 2007 | 3,956 | 1,627 | 2,329 | 146 | 654 | 2,939 | 197 |
| 2008 | 3,770 | 1,576 | 2,194 | 141 | 581 | 2,853 | 180 |
| 2009 | 3,931 | 1,631 | 2,300 | 159 | 621 | 2,892 | 250 |
| 2010 | 3,875 | 1,539 | 2,336 | 158 | 606 | 2,861 | 235 |
| 2011 | 3,884 | 1,542 | 2,342 | 159 | 631 | 2,849 | 231 |
| 2012 | 3,903 | 1,547 | 2,356 | 182 | 580 | 2,906 | 221 |
| 2013 | 4,057 | 1,702 | 2,355 | 164 | 686 | 2,956 | 235 |
| 2014 | 4,355 | 1,764 | 2,591 | 197 | 735 | 3,159 | 249 |
| 2015 | 4,428 | 1,859 | 2,569 | 190 | 711 | 3,217 | 289 |
| 2016 | 4,591 | 1,884 | 2,707 | 185 | 770 | 3,319 | 295 |
| 2017 | 4,837 | 2,061 | 2,776 | 254 | 807 | 3,473 | 283 |
| 2018 | 5,104 | 2,167 | 2,937 | 238 | 860 | 3,618 | 369 |
| 2019 | 5,363 | 2,259 | 3,104 | 253 | 950 | 3,755 | 379 |
| 2020 | 5,133 | 2,117 | 3,016 | 239 | 921 | 3,581 | 365 |
| 2021 | 5,696 | 2,310 | 3,386 | 251 | 990 | 4,009 | 409 |
| 2022 | 5,772 | 2,436 | 3,336 | 221 | 1,017 | 4,084 | 409 |
| 2023 | 6,076 | 2,553 | 3,523 | 252 | 1,120 | 4,219 | 445 |
| 2024 | 6,196 | 2,672 | 3,524 | 262 | 1,146 | 4,238 | 493 |

NH, non-Hispanic

**Supplemental Table 2** Aortic Dissection–related Mortality, Stratified by Place of Death in Adults in the United States, 1999 to 2024.

| Place of Death | Deaths |
| --- | --- |
| Medical Facility - Inpatient | 63,772 |
| Medical Facility - Outpatient or ER | 23,927 |
| Medical Facility - Dead on Arrival | 1,177 |
| Medical Facility - Status unknown | 100 |
| Decedent's home | 16,845 |
| Hospice facility | 2,197 |
| Nursing home/long term care | 2,995 |
| Other | 4,153 |
| Place of death unknown | 282 |
| Total | 115,448 |

**Supplemental Table 3** Annual percent change (APC) of Aortic Dissection–related Age-Adjusted Mortality Rates per 100,000 in Adults in the United States, 1999 to 2024.

| Year Interval | APC (95% CI) |
| --- | --- |
| Overall |  |
| 1999-2012 | −1.45 (−1.89 to −1.07) |
| 2012-2024 | 2.56 (2.16 to 3.08) |
| Female |  |
| 1999-2012 | −1.35 (−1.92 to −0.83) |
| 2012-2024 | 2.78 (2.29 to 3.39) |
| Male |  |
| 1999-2013 | −1.58 (−2.01 to −1.21) |
| 2013-2024 | 2.57 (2.09 to 3.16) |
| Hispanic or Latino |  |
| 1999-2013 | −2.04 (−4.09 to −0.72) |
| 2013-2024 | 3.12 (1.83 to 5.8) |
| NH Asian or Pacific Islander | |
| 1999-2024 | −0.63 (−1.19 to 0.03) |
| NH Black or African American | |
| 1999-2006 | 1.3 (−0.63 to 7.51) |
| 2006-2012 | −2.48 (−7.84 to 7.65) |
| 2012-2024 | 3.81 (1.28 to 5.94) |
| NH White |  |
| 1999-2012 | −1.52 (−1.99 to −1.1) |
| 2012-2024 | 2.48 (2.02 to 3.03) |
| Northeast |  |
| 1999-2013 | −1.44 (−2.56 to −0.69) |
| 2013-2024 | 2.54 (1.56 to 4.45) |
| Midwest |  |
| 1999-2011 | −1.44 (−2.32 to −0.79) |
| 2011-2024 | 2.46 (1.91 to 3.22) |
| South |  |
| 1999-2003 | 1.47 (−1.63 to 8.75) |
| 2003-2012 | −2.5 (−6.38 to 4.91) |
| 2012-2024 | 3.24 (2.17 to 4.44) |
| West |  |
| 1999-2012 | −1.58 (−3.26 to −0.64) |
| 2012-2024 | 2.37 (1.39 to 3.95) |
| Metropolitan |  |
| 1999-2012 | −1.48 (−2.13 to −0.98) |
| 2012-2020 | 2.06 (1.2 to 3.61) |
| Non-metropolitan |  |
| 1999-2012 | −1.21 (−2.26 to −0.56) |
| 2012-2020 | 2.93 (1.63 to 5.5) |
| Age 25-44 years |  |
| 1999-2017 | 1.32 (−1.23 to 3.15) |
| 2017-2024 | 3.2 (1.56 to 8.88) |
| Age 45-64 years |  |
| 1999-2012 | −0.4 (−1.41 to 0.25) |
| 2012-2024 | 3.03 (2.4 to 4.08) |
| Age 65+ years |  |
| 1999-2013 | −2.31 (−2.9 to −1.82) |
| 2013-2024 | 2.17 (1.56 to 2.98) |

NH, non-Hispanic

**Supplemental Table 4** Overall and Sex‐Stratified Aortic Dissection–related Age-Adjusted Mortality Rates per 100,000 in Adults in the United States, 1999 to 2024.

| Year | Overall | Female | Male |
| --- | --- | --- | --- |
| 1999 | 2.1 (2.04-2,17) | 1.5 (1.43-1.58) | 2.81 (2.69-2.93) |
| 2000 | 2.12 (2.06-2.19) | 1.5 (1.42-1.57) | 2.89 (2.77-3.01) |
| 2001 | 2.04 (1.97-2.1) | 1.46 (1.39-1.54) | 2.77 (2.65-2.89) |
| 2002 | 2.01 (1.95-2.07) | 1.42 (1.35-1.49) | 2.74 (2.62-2.85) |
| 2003 | 2.1 (2.03-2.16) | 1.47 (1.4-1.54) | 2.84 (2.72-2.96) |
| 2004 | 1.95 (1.89-2.02) | 1.41 (1.34-1.48) | 2.56 (2.45-2.67) |
| 2005 | 1.96 (1.89-2.02) | 1.36 (1.3-1.43) | 2.64 (2.53-2.74) |
| 2006 | 1.98 (1.92-2.05) | 1.45 (1.38-1.53) | 2.57 (2.46-2.68) |
| 2007 | 1.94 (1.88-2) | 1.4 (1.33-1.47) | 2.55 (2.44-2.65) |
| 2008 | 1.82 (1.76-1.88) | 1.33 (1.27-1.4) | 2.35 (2.25-2.45) |
| 2009 | 1.89 (1.83-1.95) | 1.38 (1.31-1.45) | 2.43 (2.33-2.54) |
| 2010 | 1.82 (1.76-1.87) | 1.28 (1.21-1.34) | 2.42 (2.32-2.52) |
| 2011 | 1.77 (1.72-1.83) | 1.27 (1.21-1.34) | 2.35 (2.25-2.45) |
| 2012 | 1.78 (1.72-1.83) | 1.24 (1.18-1.31) | 2.35 (2.25-2.45) |
| 2013 | 1.83 (1.77-1.88) | 1.33 (1.26-1.39) | 2.3 (2.2-2.39) |
| 2014 | 1.86 (1.8-1.92) | 1.33 (1.27-1.39) | 2.46 (2.37-2.56) |
| 2015 | 1.89 (1.83-1.95) | 1.37 (1.31-1.44) | 2.39 (2.29-2.48) |
| 2016 | 1.92 (1.86-1.97) | 1.39 (1.33-1.46) | 2.51 (2.41-2.61) |
| 2017 | 1.98 (1.92-2.04) | 1.47 (1.4-1.53) | 2.49 (2.4-2.59) |
| 2018 | 2.04 (1.99-2.1) | 1.54 (1.47-1.61) | 2.56 (2.46-2.65) |
| 2019 | 2.13 (2.07-2.18) | 1.57 (1.5-1.64) | 2.71 (2.61-2.81) |
| 2020 | 2.02 (1.96-2.08) | 1.47 (1.4-1.53) | 2.61 (2.51-2.71) |
| 2021 | 2.28 (2.22-2.34) | 1.65 (1.58-1.72) | 2.96 (2.86-3.06) |
| 2022 | 2.28 (2.22-2.34) | 1.65 (1.59-1.72) | 2.88 (2.78-2.98) |
| 2023 | 2.36 (2.3-2.43) | 1.73 (1.66-1.8) | 2.99 (2.89-3.09) |
| 2024 | 2.41 (2.35-2.47) | 1.81 (1.74-1.88) | 2.99 (2.89-3.09) |

**Supplemental Table 5** Aortic Dissection–related Age-Adjusted Mortality Rates per 100,000, Stratified by Race in Adults in the United States, 1999 to 2024.

| Year | Hispanic/ Latino | NH Asian/ Pacific Islander | NH Black/ African American | NH White |
| --- | --- | --- | --- | --- |
| 1999 | 1.34 (1.1-1.58) | 1.84 (1.45-2.29) | 2.89 (2.63-3.15) | 2.05 (1.98-2.12) |
| 2000 | 1.58 (1.33-1.83) | 2.43 (1.96-2.91) | 2.92 (2.66-3.18) | 2.08 (2.01-2.16) |
| 2001 | 1.24 (1.03-1.46) | 2.62 (2.15-3.1) | 2.68 (2.43-2.92) | 1.99 (1.92-2.06) |
| 2002 | 1.31 (1.09-1.53) | 1.63 (1.29-2.03) | 2.85 (2.6-3.1) | 1.99 (1.92-2.07) |
| 2003 | 1.33 (1.12-1.54) | 1.96 (1.59-2.34) | 3.04 (2.78-3.29) | 2.03 (1.96-2.11) |
| 2004 | 1.23 (1.04-1.42) | 1.79 (1.45-2.14) | 2.88 (2.63-3.12) | 1.88 (1.81-1.95) |
| 2005 | 1.23 (1.04-1.42) | 2.25 (1.86-2.63) | 3.03 (2.78-3.28) | 1.87 (1.8-1.93) |
| 2006 | 1.29 (1.1-1.48) | 1.92 (1.57-2.26) | 3.17 (2.92-3.42) | 1.9 (1.83-1.97) |
| 2007 | 1.19 (1.01-1.37) | 1.98 (1.65-2.32) | 3.1 (2.85-3.34) | 1.86 (1.79-1.93) |
| 2008 | 1.04 (0.88-1.21) | 1.89 (1.57-2.21) | 2.72 (2.49-2.95) | 1.78 (1.72-1.85) |
| 2009 | 1.34 (1.16-1.52) | 1.96 (1.64-2.28) | 2.88 (2.65-3.11) | 1.79 (1.72-1.85) |
| 2010 | 1.18 (1.02-1.35) | 1.91 (1.6-2.22) | 2.72 (2.5-2.95) | 1.76 (1.69-1.82) |
| 2011 | 1.11 (0.96-1.26) | 1.79 (1.5-2.07) | 2.79 (2.57-3.01) | 1.7 (1.64-1.76) |
| 2012 | 1 (0.86-1.14) | 1.9 (1.62-2.19) | 2.51 (2.3-2.72) | 1.74 (1.67-1.8) |
| 2013 | 1.08 (0.93-1.22) | 1.6 (1.35-1.86) | 2.85 (2.63-3.07) | 1.74 (1.68-1.81) |
| 2014 | 1.07 (0.93-1.21) | 1.8 (1.54-2.06) | 3.04 (2.82-3.27) | 1.82 (1.75-1.89) |
| 2015 | 1.18 (1.04-1.32) | 1.67 (1.43-1.91) | 2.88 (2.67-3.1) | 1.81 (1.74-1.87) |
| 2016 | 1.16 (1.02-1.3) | 1.58 (1.35-1.82) | 3.04 (2.82-3.26) | 1.88 (1.82-1.95) |
| 2017 | 1.06 (0.93-1.19) | 2.01 (1.76-2.27) | 3.11 (2.89-3.33) | 1.91 (1.85-1.98) |
| 2018 | 1.32 (1.18-1.46) | 1.8 (1.57-2.04) | 3.27 (3.05-3.5) | 1.99 (1.92-2.06) |
| 2019 | 1.36 (1.22-1.5) | 1.85 (1.62-2.08) | 3.57 (3.34-3.8) | 2.01 (1.95-2.08) |
| 2020 | 1.21 (1.08-1.34) | 1.68 (1.46-1.89) | 3.4 (3.18-3.62) | 1.94 (1.88-2.01) |
| 2021 | 1.35 (1.21-1.49) | 2.02 (1.78-2.26) | 3.67 (3.43-3.9) | 2.22 (2.15-2.29) |
| 2022 | 1.35 (1.21-1.48) | 1.67 (1.46-1.88) | 3.79 (3.55-4.03) | 2.21 (2.14-2.29) |
| 2023 | 1.42 (1.29-1.56) | 1.8 (1.58-2.01) | 4.12 (3.87-4.37) | 2.27 (2.2-2.34) |
| 2024 | 1.56 (1.42-1.7) | 1.85 (1.64-2.07) | 4.16 (3.91-4.41) | 2.28 (2.21-2.35) |

NH, non-Hispanic

**Supplemental Table 6** Aortic Dissection–related Crude Mortality Rates per 100,000, Stratified by Age Group in Adults in the United States, 1999 to 2024.

| Year | Age 25-44 years | 45-64 years | 65+ years |
| --- | --- | --- | --- |
| 1999 | 0.42 (0.38-0.47) | 1.77 (1.66-1.87) | 6.63 (6.36-6.9) |
| 2000 | 0.39 (0.35-0.43) | 1.77 (1.67-1.88) | 6.85 (6.58-7.13) |
| 2001 | 0.43 (0.39-0.47) | 1.62 (1.52-1.72) | 6.58 (6.31-6.85) |
| 2002 | 0.39 (0.35-0.43) | 1.74 (1.64-1.84) | 6.39 (6.12-6.65) |
| 2003 | 0.42 (0.38-0.47) | 1.81 (1.71-1.91) | 6.56 (6.3-6.83) |
| 2004 | 0.45 (0.4-0.49) | 1.56 (1.47-1.66) | 6.17 (5.92-6.43) |
| 2005 | 0.45 (0.4-0.49) | 1.76 (1.66-1.85) | 6.01 (5.76-6.26) |
| 2006 | 0.46 (0.42-0.51) | 1.78 (1.68-1.87) | 5.99 (5.74-6.24) |
| 2007 | 0.49 (0.45-0.54) | 1.77 (1.68-1.87) | 5.77 (5.52-6.01) |
| 2008 | 0.44 (0.4-0.49) | 1.63 (1.54-1.72) | 5.49 (5.25-5.72) |
| 2009 | 0.48 (0.44-0.53) | 1.65 (1.56-1.74) | 5.58 (5.34-5.81) |
| 2010 | 0.49 (0.45-0.54) | 1.63 (1.54-1.72) | 5.32 (5.09-5.54) |
| 2011 | 0.45 (0.41-0.5) | 1.68 (1.59-1.77) | 5.13 (4.91-5.34) |
| 2012 | 0.46 (0.41-0.51) | 1.68 (1.59-1.76) | 4.94 (4.73-5.15) |
| 2013 | 0.51 (0.46-0.55) | 1.65 (1.57-1.74) | 5.06 (4.85-5.27) |
| 2014 | 0.46 (0.41-0.51) | 1.85 (1.76-1.94) | 5.24 (5.03-5.45) |
| 2015 | 0.53 (0.48-0.58) | 1.78 (1.69-1.87) | 5.21 (5-5.41) |
| 2016 | 0.52 (0.48-0.57) | 1.91 (1.82-2) | 5.15 (4.95-5.35) |
| 2017 | 0.5 (0.45-0.55) | 1.97 (1.87-2.06) | 5.4 (5.2-5.6) |
| 2018 | 0.53 (0.48-0.58) | 1.97 (1.88-2.07) | 5.7 (5.49-5.9) |
| 2019 | 0.58 (0.53-0.63) | 2.07 (1.97-2.16) | 5.79 (5.59-5.99) |
| 2020 | 0.58 (0.53-0.63) | 2.03 (1.94-2.13) | 5.28 (5.09-5.47) |
| 2021 | 0.59 (0.54-0.64) | 2.16 (2.06-2.26) | 6.18 (5.97-6.39) |
| 2022 | 0.58 (0.52-0.63) | 2.34 (2.23-2.44) | 5.9 (5.7-6.1) |
| 2023 | 0.63 (0.58-0.68) | 2.33 (2.23-2.44) | 6.21 (6-6.41) |
| 2024 | 0.65 (0.6-0.71) | 2.31 (2.21-2.42) | 6.4 (6.19-6.6) |

**Supplemental Table 7** Aortic Dissection–related Age-Adjusted Mortality Rates per 100,000, Stratified by Census Region in Adults in the United States, 1999 to 2024.

| Census Region | Year | Age Adjusted Rate |
| --- | --- | --- |
| Northeast | 1999 | 2 (1.85-2.14) |
| Northeast | 2000 | 1.87 (1.73-2.01) |
| Northeast | 2001 | 1.82 (1.68-1.95) |
| Northeast | 2002 | 1.92 (1.78-2.06) |
| Northeast | 2003 | 1.93 (1.79-2.07) |
| Northeast | 2004 | 1.78 (1.65-1.92) |
| Northeast | 2005 | 1.93 (1.79-2.07) |
| Northeast | 2006 | 1.85 (1.71-1.98) |
| Northeast | 2007 | 1.86 (1.73-2) |
| Northeast | 2008 | 1.69 (1.56-1.82) |
| Northeast | 2009 | 1.79 (1.66-1.92) |
| Northeast | 2010 | 1.69 (1.57-1.82) |
| Northeast | 2011 | 1.58 (1.46-1.7) |
| Northeast | 2012 | 1.68 (1.55-1.8) |
| Northeast | 2013 | 1.55 (1.43-1.67) |
| Northeast | 2014 | 1.71 (1.58-1.84) |
| Northeast | 2015 | 1.7 (1.58-1.82) |
| Northeast | 2016 | 1.7 (1.58-1.83) |
| Northeast | 2017 | 1.79 (1.66-1.91) |
| Northeast | 2018 | 1.72 (1.6-1.84) |
| Northeast | 2019 | 1.97 (1.84-2.1) |
| Northeast | 2020 | 1.94 (1.8-2.07) |
| Northeast | 2021 | 2.05 (1.92-2.19) |
| Northeast | 2022 | 1.92 (1.8-2.05) |
| Northeast | 2023 | 2.15 (2.01-2.29) |
| Northeast | 2024 | 2.12 (1.99-2.26) |
| Midwest | 1999 | 2.4 (2.25-2.55) |
| Midwest | 2000 | 2.25 (2.1-2.39) |
| Midwest | 2001 | 2.21 (2.06-2.35) |
| Midwest | 2002 | 1.94 (1.81-2.07) |
| Midwest | 2003 | 2.17 (2.03-2.31) |
| Midwest | 2004 | 1.98 (1.85-2.11) |
| Midwest | 2005 | 2.17 (2.03-2.3) |
| Midwest | 2006 | 2.05 (1.92-2.18) |
| Midwest | 2007 | 2.03 (1.9-2.16) |
| Midwest | 2008 | 1.98 (1.85-2.11) |
| Midwest | 2009 | 2.05 (1.92-2.18) |
| Midwest | 2010 | 1.85 (1.73-1.97) |
| Midwest | 2011 | 1.88 (1.76-2) |
| Midwest | 2012 | 1.95 (1.83-2.08) |
| Midwest | 2013 | 2.06 (1.93-2.19) |
| Midwest | 2014 | 2.11 (1.98-2.24) |
| Midwest | 2015 | 2.06 (1.93-2.18) |
| Midwest | 2016 | 2.11 (1.98-2.24) |
| Midwest | 2017 | 2.28 (2.14-2.41) |
| Midwest | 2018 | 2.29 (2.16-2.42) |
| Midwest | 2019 | 2.31 (2.17-2.44) |
| Midwest | 2020 | 2.24 (2.11-2.37) |
| Midwest | 2021 | 2.43 (2.29-2.56) |
| Midwest | 2022 | 2.53 (2.39-2.67) |
| Midwest | 2023 | 2.57 (2.43-2.71) |
| Midwest | 2024 | 2.59 (2.45-2.73) |
| South | 1999 | 1.92 (1.81-2.03) |
| South | 2000 | 2 (1.89-2.11) |
| South | 2001 | 1.88 (1.78-1.99) |
| South | 2002 | 2.1 (1.98-2.21) |
| South | 2003 | 2.09 (1.98-2.2) |
| South | 2004 | 2 (1.89-2.1) |
| South | 2005 | 1.82 (1.72-1.92) |
| South | 2006 | 1.9 (1.8-2) |
| South | 2007 | 1.83 (1.73-1.93) |
| South | 2008 | 1.75 (1.66-1.85) |
| South | 2009 | 1.8 (1.7-1.89) |
| South | 2010 | 1.73 (1.64-1.82) |
| South | 2011 | 1.67 (1.58-1.76) |
| South | 2012 | 1.6 (1.51-1.69) |
| South | 2013 | 1.75 (1.66-1.85) |
| South | 2014 | 1.78 (1.69-1.87) |
| South | 2015 | 1.77 (1.68-1.86) |
| South | 2016 | 1.84 (1.74-1.93) |
| South | 2017 | 1.83 (1.74-1.92) |
| South | 2018 | 1.91 (1.81-2) |
| South | 2019 | 2.05 (1.96-2.15) |
| South | 2020 | 1.88 (1.79-1.97) |
| South | 2021 | 2.23 (2.13-2.33) |
| South | 2022 | 2.25 (2.15-2.34) |
| South | 2023 | 2.31 (2.21-2.41) |
| South | 2024 | 2.43 (2.33-2.53) |
| West | 1999 | 2.22 (2.07-2.38) |
| West | 2000 | 2.49 (2.33-2.66) |
| West | 2001 | 2.37 (2.22-2.53) |
| West | 2002 | 2.16 (2.01-2.31) |
| West | 2003 | 2.2 (2.05-2.35) |
| West | 2004 | 1.87 (1.74-2) |
| West | 2005 | 2.05 (1.91-2.19) |
| West | 2006 | 2.17 (2.03-2.32) |
| West | 2007 | 2.13 (1.99-2.27) |
| West | 2008 | 1.92 (1.79-2.05) |
| West | 2009 | 1.89 (1.76-2.01) |
| West | 2010 | 2.01 (1.88-2.14) |
| West | 2011 | 1.99 (1.86-2.12) |
| West | 2012 | 1.86 (1.74-1.99) |
| West | 2013 | 1.85 (1.73-1.97) |
| West | 2014 | 1.95 (1.83-2.07) |
| West | 2015 | 2.08 (1.95-2.2) |
| West | 2016 | 2.13 (2-2.25) |
| West | 2017 | 2.12 (2-2.24) |
| West | 2018 | 2.32 (2.19-2.44) |
| West | 2019 | 2.25 (2.13-2.38) |
| West | 2020 | 2.18 (2.05-2.3) |
| West | 2021 | 2.46 (2.33-2.59) |
| West | 2022 | 2.33 (2.21-2.46) |
| West | 2023 | 2.47 (2.34-2.6) |
| West | 2024 | 2.42 (2.3-2.55) |

**Supplemental Table 8** Aortic Dissection–related Age-Adjusted Mortality Rates per 100,000, Stratified by Urban-Rural Classification in Adults in the United States, 1999 to 2020.

| Year | Metropolitan | Non-metropolitan |
| --- | --- | --- |
| 1999 | 2.12 (2.05-2.19) | 2.03 (1.88-2.19) |
| 2000 | 2.22 (2.14-2.29) | 1.89 (1.74-2.04) |
| 2001 | 2.07 (2-2.14) | 1.87 (1.73-2.02) |
| 2002 | 2.01 (1.94-2.08) | 1.89 (1.74-2.04) |
| 2003 | 2.12 (2.04-2.19) | 2.09 (1.94-2.25) |
| 2004 | 1.97 (1.9-2.04) | 1.8 (1.66-1.94) |
| 2005 | 2 (1.93-2.07) | 1.85 (1.7-1.99) |
| 2006 | 1.99 (1.92-2.06) | 1.96 (1.82-2.11) |
| 2007 | 1.96 (1.89-2.02) | 1.87 (1.73-2.02) |
| 2008 | 1.86 (1.79-1.92) | 1.75 (1.61-1.89) |
| 2009 | 1.9 (1.83-1.96) | 1.8 (1.66-1.94) |
| 2010 | 1.84 (1.78-1.91) | 1.69 (1.55-1.83) |
| 2011 | 1.78 (1.72-1.84) | 1.69 (1.55-1.83) |
| 2012 | 1.79 (1.72-1.85) | 1.68 (1.54-1.81) |
| 2013 | 1.82 (1.76-1.88) | 1.78 (1.65-1.92) |
| 2014 | 1.88 (1.82-1.94) | 1.75 (1.62-1.89) |
| 2015 | 1.89 (1.83-1.95) | 1.91 (1.76-2.05) |
| 2016 | 1.93 (1.87-1.99) | 1.92 (1.78-2.07) |
| 2017 | 2 (1.94-2.06) | 1.96 (1.81-2.1) |
| 2018 | 2.03 (1.96-2.09) | 2.08 (1.93-2.23) |
| 2019 | 2.13 (2.07-2.19) | 2.1 (1.95-2.25) |
| 2020 | 2.04 (1.98-2.1) | 2.1 (1.95-2.26) |

**Supplemental Table 9** Aortic Dissection–related Age-Adjusted Mortality Rates per 100,000, Stratified by State in Adults in the United States, 1999 to 2020.

| State | Age Adjusted Rate (95% CI) |
| --- | --- |
| Alabama | 1.86 (1.76-1.96) |
| Alaska | 2.14 (1.78-2.49) |
| Arizona | 1.94 (1.85-2.02) |
| Arkansas | 1.73 (1.61-1.85) |
| California | 2.05 (2.01-2.09) |
| Colorado | 2.11 (2-2.21) |
| Connecticut | 1.68 (1.57-1.78) |
| Delaware | 2.35 (2.1-2.61) |
| District of Columbia | 3.01 (2.65-3.38) |
| Florida | 1.81 (1.77-1.86) |
| Georgia | 1.99 (1.92-2.07) |
| Hawaii | 3.12 (2.89-3.35) |
| Idaho | 1.79 (1.62-1.97) |
| Illinois | 1.89 (1.83-1.96) |
| Indiana | 2.14 (2.05-2.24) |
| Iowa | 2.08 (1.95-2.21) |
| Kansas | 2.23 (2.09-2.37) |
| Kentucky | 1.64 (1.54-1.74) |
| Louisiana | 1.81 (1.71-1.91) |
| Maine | 1.68 (1.51-1.85) |
| Maryland | 1.98 (1.89-2.08) |
| Massachusetts | 1.45 (1.38-1.53) |
| Michigan | 2.41 (2.33-2.48) |
| Minnesota | 2.09 (1.99-2.19) |
| Mississippi | 1.55 (1.43-1.67) |
| Missouri | 1.93 (1.84-2.02) |
| Montana | 2.07 (1.85-2.3) |
| Nebraska | 1.94 (1.78-2.1) |
| Nevada | 2.15 (2-2.3) |
| New Hampshire | 1.71 (1.54-1.89) |
| New Jersey | 1.65 (1.58-1.71) |
| New Mexico | 1.86 (1.7-2.01) |
| New York | 1.89 (1.84-1.94) |
| North Carolina | 1.86 (1.79-1.93) |
| North Dakota | 1.81 (1.56-2.06) |
| Ohio | 2.19 (2.12-2.26) |
| Oklahoma | 2.02 (1.9-2.13) |
| Oregon | 2.15 (2.03-2.27) |
| Pennsylvania | 1.94 (1.88-2) |
| Rhode Island | 1.91 (1.71-2.12) |
| South Carolina | 2.22 (2.11-2.34) |
| South Dakota | 1.83 (1.59-2.06) |
| Tennessee | 1.99 (1.9-2.08) |
| Texas | 1.77 (1.73-1.82) |
| Utah | 2.15 (1.98-2.31) |
| Vermont | 2.16 (1.87-2.45) |
| Virginia | 1.59 (1.52-1.66) |
| Washington | 2.22 (2.13-2.32) |
| West Virginia | 1.8 (1.65-1.94) |
| Wisconsin | 2 (1.91-2.1) |
| Wyoming | 2.15 (1.84-2.46) |
